# “Pore-Like” Effects of Super-Molecular Self-Assembly on Molecular Diffusion of Poly(Ethylene Oxide)-Poly(Propylene Oxide)-Poly(Ethylene Oxide) in Water

**DOI:** 10.3390/ma5050966

**Published:** 2012-05-24

**Authors:** Konstantin Ulrich, Petrik Galvosas, Jörg Kärger, Farida Grinberg

**Affiliations:** 1 Department of Physics, Universität Leipzig, Linnstrasse 5, 04103 Leipzig, Germany; E-Mails: konstantin.ulrich@uni-leipzig.de (K.U.); petrik.galvosas@vuw.ac.nz (P.G.); kaerger@uni-leipzig.de (J.K.); 2 MacDiarmid Institute for Advanced Materials and Nanotechnology, School of Chemical and Physical Sciences, Victoria University of Wellington, Wellington 6140, New Zealand; 3 Institute of Neuroscience and Medicine-4, Forschungszentrum Jülich GmbH, 52425 Jülich, Germany

**Keywords:** anisotropic diffusion, super-molecular structure, pulsed field gradient NMR

## Abstract

Molecular diffusion of triblock copolymers poly(ethylene oxide)-poly(propylene oxide)-poly(ethylene oxide) in water was studied with the help of Pulsed Field Gradient NMR in the broad range of polymer weight fractions from 0.09 to 0.8. Owing to amphiphilic nature of the molecules, these block copolymers exhibit rich self-organization properties when mixed with water. In particular, at ambient temperatures they form micelles and three liquid crystalline mesophases: cubic, hexagonal, and lamellar. The corresponding super-molecular structure formations were studied with the same block copolymer and at the same temperature. Self-assembly of molecules was shown to produce “pore-like” effects on their self-diffusion properties by imposing severe constraints on the dimensionality of propagation. Diffusion in the hexagonal phase was shown to be quasi one-dimensional in the direction parallel to the long axis of the ordered molecular rods. In the lamellar phase, diffusion was found to be quasi two-dimensional, in the plane of the lamellar structures. The observed diffusion anisotropy was attributed to the effects of the specific molecular ordering on the mesoscopic length scale.

## 1. Introduction

Recent explosion of interest to block copolymers (BCP) was caused by their fascinating properties of self-assembly and spontaneous structure formation on the mesoscopic length scale. During the last two decades, these properties found widespread applications in drug delivery, gene therapy, nanotechnology and in molecular engineering [1,2,3,4,5]. Self-association of molecules is central also for understanding biological functions. One of the most significant BCPs are represented by symmetric triblock copolymers based on poly(ethylene oxide) (PEO) and poly(propylene oxide) (PPO). These substances are also known as Poloxamers or Pluronics (the trade name by BASF Corp.). Owing to the amphiphilic character of molecules, Pluronics exhibit a remarkable variety of super-molecular structures when mixed with water [6,7]. Due to their rich lyotropic and thermotropic properties, Pluronics represent good models for investigation of self-organizing systems.

Self-assembly of PEO-PPO-PEO copolymers is caused by the different solubility of the PEO and PPO blocks in water. While water is a good solvent for PEO in broad temperature and concentration ranges, solubility of the PPO block in water is limited to the temperatures below the ambient temperatures and to small polymer concentrations. This gives rise to dramatic temperature and concentration dependences of structure and dynamic properties of Pluronics. At low polymer concentrations and low temperatures, PEO-PPO-PEO molecules dissolve in water as individual Gaussian chains. With increasing temperature or polymer concentration above the critical micellar temperature CMT and the critical micellar concentration CMC, they form spherical micellar aggregates consisting of relatively compact cores of the PPO blocks surrounded by hydrated coronas of the PEO blocks [7,8]. At temperatures above CMT, further increase of the polymer concentration gives rise to the formation of the cubic mesophases [9]. This transition is induced by close-packing of spherical micelles governed by entropic forces. The cubic phase exhibits gel-like properties and its occurrence was reported for the large variety of PEO-PPO-PEO copolymers. At even higher polymer concentrations, additional liquid crystalline mesophases (hexagonal and lamellar) may form. In contrast to the isotropic liquid micellar and cubic micellar phases, the latter mesophases are characteristic of anisotropic molecular ordering on the mesoscopic length scale [6].

Self-assembling systems including the BCPs represent an area of intensive research [6,7,8,9,10,11,12,13]. Much progress has been done in elucidation of their structure characteristics with the help of small-angle neutron scattering (SANS) [9,13]. However, molecular dynamical properties and their relationship with the underlying microstructure remain only poorly understood. This is, in particular, due to the fact that a formation of the super-molecular structures gives rise to very slow dynamical processes not easily accessible by conventional techniques. The Pulsed Field Gradient (PFG) NMR provides the powerful means of direct diffusion measurements of fluids [14,15]. However, only a small number of works were devoted to the diffusion studies of BCPs using this method. The published studies were predominantly limited to diblock copolymers in melts [16,17,18] or to the micellar phases of di- and triblock copolymers [19,20] in solvents. A few experiments were related to the cubic phases [21,22]. Self-diffusion of multi-block copolymers in selective solvents was investigated in one of the earlier works [23] by Grinberg *et al*.

In general, experimental difficulties of measuring low macromolecular diffusivities in viscous solutions and melts using PFG NMR are related to the requirements on the field gradient system representing a significant technical challenge. Recently, using a home-built system with ultra-high-intensity field gradient pulses we demonstrated [24,25] the feasibility of measuring the diffusivities of triblock PEO-PPO-PEO copolymers mixed with water in bulk systems and confined in nanochannels of mesoporous material of type SBA-15. The measured diffusivities were shown to cover remarkable 4 orders of magnitude at the same temperature. The aim of the present study was a more detailed investigation of the effects of super-molecular organization on self-diffusion of PEO-PPO-PEO in water. This was achieved by monitoring diffusion of the two Pluronic samples in a broad range of polymer concentrations in which the most typical super-molecular structures (micellar, cubic, hexagonal and lamellar) could be observed. A special accent was put on understanding the influence of self-assembly on diffusion anisotropy.

## 2. Experimental Section

**Figure 1:**
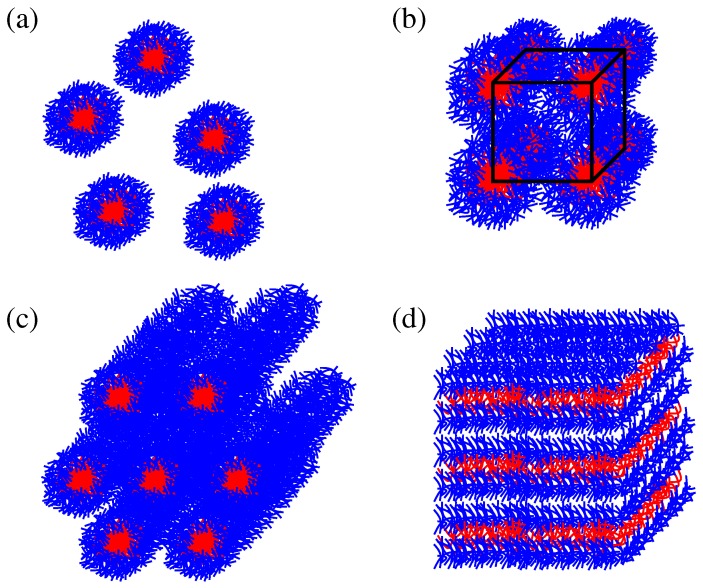
Schematic drawings of the micellar (**a**), cubic (**b**), hexagonal (**c**) and lamellar (**d**) structures of Pluronic BCPs in selective solvents.

Samples of (PEO)${}_{27}$-(PPO)${}_{61}$-(PEO)${}_{27}$ (trade name Pluronic P104) and (PEO)${}_{20}$-(PPO)${}_{70}$-(PEO)${}_{20}$ (trade name Pluronic P123) were obtained as a gift from BASF Corp. (New Jersey) and used without further purification. According to the manufacturer, the nominal molecular masses MM of the Pluronic P104 and Pluronic P123 are ∼5900 g mol${}^{-1}$ and ∼5750 g mol${}^{-1}$, respectively. In the following, Pluronic P104 and Pluronic 123 will be denoted as P104 and P123 for simplicity. The homopolymer poly(ethylene oxide) (PEO) with a nominal MM of ∼6000 g mol${}^{-1}$ was purchased from Sigma-Aldrich (Taufkirchen, Germany). Deuterated water used as solvent was purchased from Chemotrade (99.98%, Leipzig, Germany). In mixtures with water, the BCPs under study form typical super-molecular structures [6]. Schematic drawings of these microstructures are shown in Figure 1. The investigated range of the polymer mass fractions in water, *c*, was between 0.09 and 0.8 (concentrations *c* are dimensionless weight-to-weight fractions in this article). At room temperature, the investigated Pluronics exhibit in this concentration range the micellar, cubic, hexagonal and lamellar phases [6,26,27]. (The expression “micellar phase” refers to the “micellar disordered” or “micellar liquid” phase; the “cubic phase” denotes micellar phase arranged with the three-dimensional cubatic order). Two Pluronic samples, P123 and P104, with similar phase properties were selected in order to elucidate the characteristic properties of the distinguished phases which are not sensitive to the small variations in the block composition. Table 1 shows the critical concentrations of the phase transitions of interest for these samples at $T=25{\;}^{\circ }$C.

The investigated samples at various polymer concentrations were prepared by weighing the components. For the PFG NMR measurements the samples were filled into special NMR glass tubes with an outer diameter of 7.5 mm. The tubes were then sealed and stored for at least 24 h in a refrigerator at $5{\;}^{\circ }$C for reaching homogeneity. The homogeneity was controlled by visual inspection.

**Table 1:** Phase transition temperatures and concentrations of studied samples at $25{\;}^{\circ }$C. The data on critical concentrations for P104 and P123 are given according to [26,27], respectively. $c_{\text{mic}.\to \text{cub}.}$, $c_{\text{cub}.\to \text{hex}.}$, $c_{\text{hex}.\to 2\Phi }$, and $c_{2\Phi \to \text{lam}.}$ denote, respectively, the transition concentrations from the micellar to cubic, from the cubic to hexagonal, from the hexagonal to multiphase (2Φ), and from the multiphase to the lamellar state. Very narrow multiphase concentration regions separating the micellar, cubic and the hexagonal phases were disregarded. The transition temperature for P123 was taken form [6].

polymer	$c_{\text{mic}.\to \text{cub}.}$	$c_{\text{cub}.\to \text{hex}.}$	$c_{\text{hex}.\to 2\Phi }$	$c_{2\Phi \to \text{lam}.}$	$T_{\text{cub}.\to \text{mic}./\text{uni}.}$
	through 2Φ	through 2Φ			at c=0.29
P104	≈0.28	≈0.43	0.63	0.70	
P123	≈0.25	≈0.36	0.53	0.68	$\approx \;17{\;}^{\circ }$C

Diffusion measurements were performed with the home-made PFG NMR diffusometer FEGRIS 400 operating at a proton resonance frequency of 400 MHz. The description of the hardware and the details of the method can be found elsewhere [28,29]. The three ninety-degree pulse $90^{\circ }-\tau_{1}-90^{\circ }-\tau_{2}-90^{\circ }-\tau_{1}-echo$ sequence (usually referred to as the “stimulated-echo pulse sequence”) was applied. In the simplest case of normal isotropic diffusion, the spin echo amplitudes at the echo time $2\tau_{1}+\tau_{2}$ are [30]

(1) $$\Psi (q,t)=\exp\left(-q^{2}tD\right)$$

with q=γδg, where *g* and *δ* are the amplitude and the duration of the magnetic-field gradient pulses, respectively, *γ* is the gyromagnetic ratio ($2.675\times 10^{8}\;T^{-1}s^{-1}$ for hydrogen), *D* is the self-diffusion coefficient. The “observation” time *t* in the “stimulated echo” pulse sequence equals $\left(∆-\frac{1}{3}\delta \right)$, where *∆* is the time interval between the first and the second magnetic field gradient pulses. The first gradient pulse is applied in the time interval between the first and the second ninety-degree pulses; the second gradient pulse is applied between the third ninety-degree pulse and the echo time. Within each experiment, the diffusion attenuation curves were measured for increasing values of *g* while the time settings of the pulse sequence were kept constant. The maximal gradient strength was 35 T/m. Typical values of *δ* were between 0.5 and 2 ms. The values of *∆* were varied in the range between 20 and 400 ms for all values of concentration.

In the case of the distribution of diffusion coefficients, the attenuation curves are given as

(2) $$\Psi (q,t)=\int P\left(D\right)\exp\left(-q^{2}tD\right)\;dD$$

where P(D) is the distribution function of diffusivities [31]. The mean diffusivity $\bar{D}$ can be evaluated from the initial slopes of the attenuation curves [31] according to

(3) $$\bar{D}=-\lim_{q^{2}t\to 0}\frac{\partial \Psi (q,t)}{\partial (q^{2}t)}=\int DP\left(D\right)\;dD$$

## 3. Results and Discussion

### 3.1. Micellar Phase

**Figure 2:**
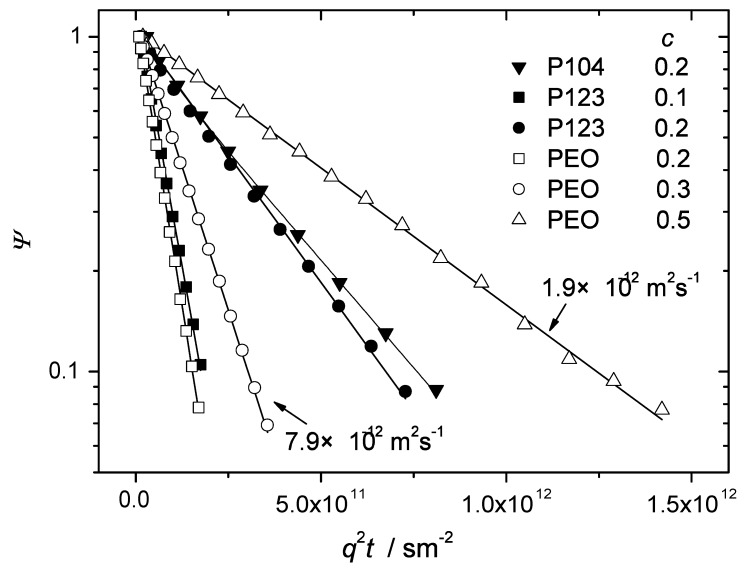
The normalized spin echo diffusion attenuations of polymer molecules in the samples P123, P104 and PEO in water at $T=23{\;}^{\circ }$C. The polymer concentrations are indicated on the plot. For the Pluronic samples these concentrations are in the range of the micellar state. All experimental data sets except for that of P104 (c=0.2) were satisfactorily fitted by mono-exponential function as shown by solid lines. The fitted values of diffusivities are indicated for two slopes as an example. The data points for P104 (c=0.2) showed deviations from the monoexponential behaviour and were therefore fitted to a biexponential function (solid curve) with the following fit parameters: ($1.56\times 10^{-11}\pm 1.7\times 10^{-12}$) m${}^{2}$s${}^{-1}$, ($2.95\times 10^{-12}\pm 3.7\times 10^{-14}$) m${}^{2}$s${}^{-1}$, and 0.17 for the fraction with the larger diffusivity.

Figure 2 shows typical diffusion attenuation curves of the investigated Pluronic samples measured at room temperature for polymer concentrations in water below or equal to 0.2. This range of concentrations is characteristic of the isotropic micellar liquid phase [6]. The attenuation curves of PEO in water solutions are shown for concentrations between 0.2 and 0.5. In PEO and P123 the observed diffusion attenuations were exponential in the dynamical range of amplitudes exceeding one order of magnitude. Any dependence on the observation time below 400 ms was below the experimental error. Thus, diffusion of PEO and P123 in the indicated range of concentration is isotropic and can be described in terms of a single diffusion coefficient *D* according to Equation (1).

**Figure 3:**
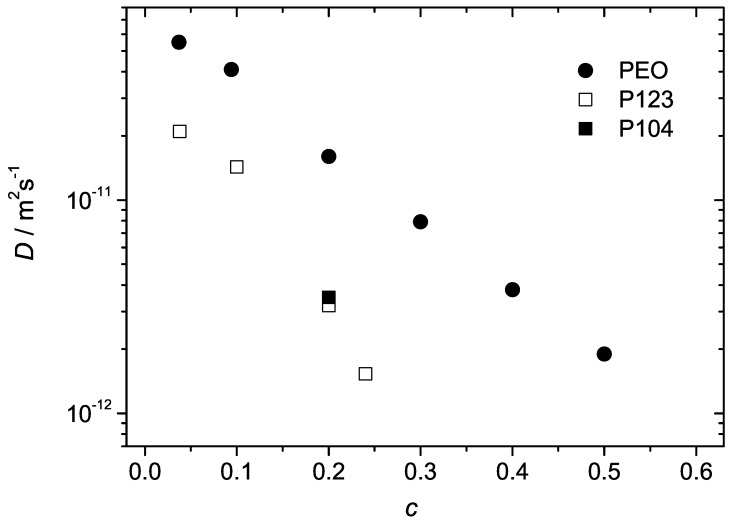
The diffusion coefficients of polymer molecules in the samples P123, P104 and PEO in water as a function of polymer concentration at $T=23{\;}^{\circ }$C. The range of concentrations for P123, P104 is limited to the micellar phase. The data points are evaluated from the spin-echo diffusion attenuations shown in Figure 2. In the case of PEO and P123 they refer to mono-exponential fits. In the case of P104 the data point was evaluated from the initial slope of the curve and represents the average diffusivity.

The solid lines in Figure 2 are exponential fits of Equation (1) to the experimental data. The fitted values of *D* are shown in Figure 3 as a function of the polymer concentration. The diffusion coefficient decreases with increasing polymer concentration as typically expected for macromolecular solutions due to increasing steric hindrances on the molecular displacements. Although the overall molecular masses of the Pluronic molecules were approximately the same as of PEO, the diffusivities of the BCPs were considerably smaller. This is obviously the effect of formation of micellar aggregates. The aggregated molecules diffuse in cooperative manner, as entire entities. The molecular mass and the hydrodynamic radius of such entities (micelles) are bigger than that of the individual PEO chains and the diffusion coefficient is therefore correspondingly smaller. For the given concentration, the values of *D* in the PEO solutions exceeded that in the BCP samples by a factor of 3 to 8. The differences became larger for larger concentrations. In P104, slight deviations from monoexponential behaviour were observed (both temperature and concentration were well above the critical values of the micelle formation [7]). The corresponding data set was fitted with the help of biexponential function. The deviations from the exponential behavior could be expected due to several reasons. First of all, the commercial Pluronics are not fractionated and might be rather polydisperse. They also may contain a certain amount of diblock copolymers (as impurities). If these factors gave rise to the distribution of micellar sizes the diffusivities of micelles were distributed as well. Besides, in BCP solutions, micelles may coexist with the dissolved unimers (individual chains not trapped in the micellar aggregates). Unimers would have larger diffusivities than that of micelles and contribute to the overall distribution function of diffusivities. In this context, the observed mono-exponentiality of the diffusion attenuations in micellar solutions of P123 is somewhat surprising. However, one can assume that heterogeneity effects on diffusion attenuations might be more or less efficiently equilibrated by fast exchange between different micelles as well as fast exchange between micelles and unimers/impurities [9] during the observation time.

The hydrodymanic radius $R_{H}$ of the micelles can be evaluated in the limit of diluted concentrations according to the Stokes–Einstein relation:

(4) $$D=\frac{k_{B}T}{6\pi \eta R_{H}}$$

where $k_{B}$ is the Boltzmann constant and *η* is the (local) viscosity.

The estimation of $R_{H}$ for P123 at $T=23{\;}^{\circ }$C based on the obtained PFG NMR data gave a value of about 9 nm (the value of diffusivity was evaluated by extrapolating the function D(c) in the range of diluted concentrations to zero). This is of the same order of magnitude as reported for similar Pluronic samples [10]. However, it exceeds the experimental value of 5.8 nm reported for P123 at $25{\;}^{\circ }$C on the basis of light scattering studies [6]. It was beyond the scope of this study to unambiguously elucidate this discrepancy in detail. However, one may attribute it to several factors: The hard sphere approximation anticipated by Equation (4) is probably too rough for being applicable to micelle aggregates. Firstly, the shape of the micelles formed in the samples with rather long hydrophobic blocks (considerably exceeding the length of the hydrophilic blocks as in our samples) is expected to be anisotropic rather than spherical [6]. Secondly, hydrated PEO-block coronas will experience a larger viscous drag force compared to the hard spheres. In addition, the differences in the applied methods (for instance light scattering methods measure the mutual diffusion coefficient, thus potentially resulting in larger diffusion coefficients and hence smaller $R_{H}$) and the potential differences in the commercial samples purchased from the different synthesis batches must also be taken into account.

### 3.2. Liquid Crystalline Cubic Phase

**Figure 4:**
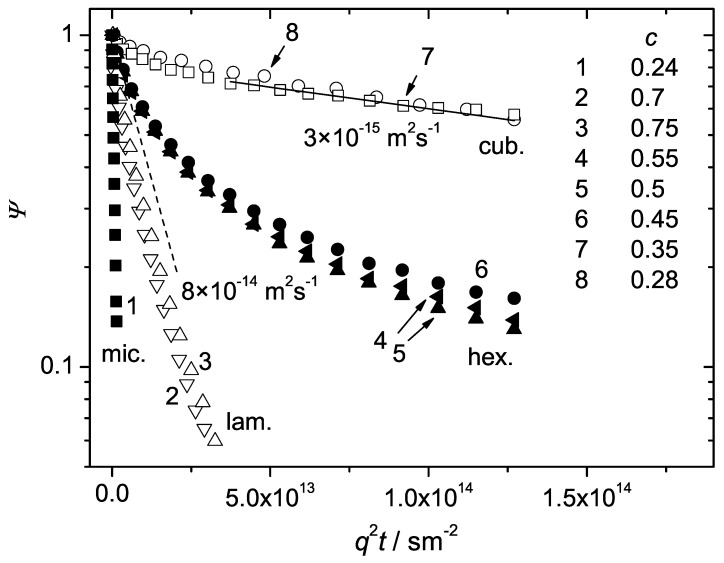
The normalized spin echo diffusion attenuations of the triblock copolymer P123 in water for different concentrations at $T=23{\;}^{\circ }$C. The polymer weight fractions for the curves 1–8 were 0.24, 0.7, 0.75, 0.55, 0.5, 0.45, 0.35 and 0.28, respectively. The solid line refers to the initial slope of the slow component. The dashed lines is the guide for the eye.

**Figure 5:**
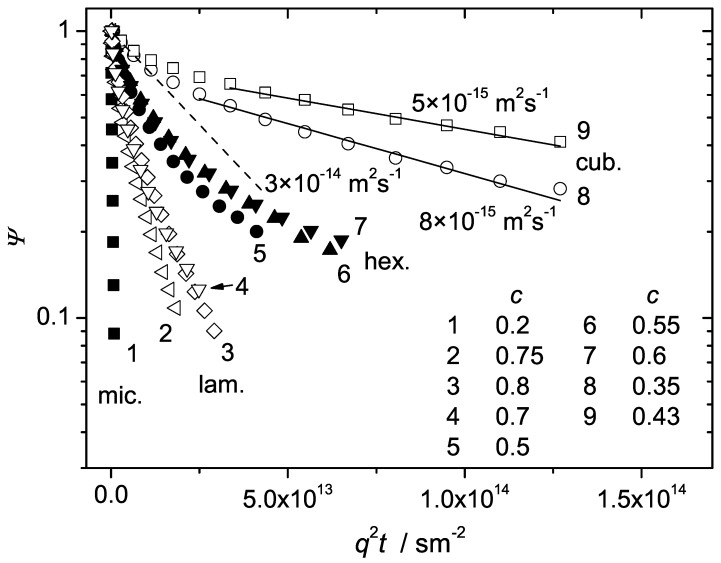
The normalized spin echo diffusion attenuations of the triblock copolymer P104 in water for different concentrations at $T=23{\;}^{\circ }$C. The polymer weight fractions for the curves 1–9 were 0.2, 0.75, 0.8, 0.7, 0.5, 0.55, 0.6, 0.35 and 0.43, respectively. The solid lines refer to the initial slopes of the slow components. The dashed line is the guide for the eye.

**Figure 6:**
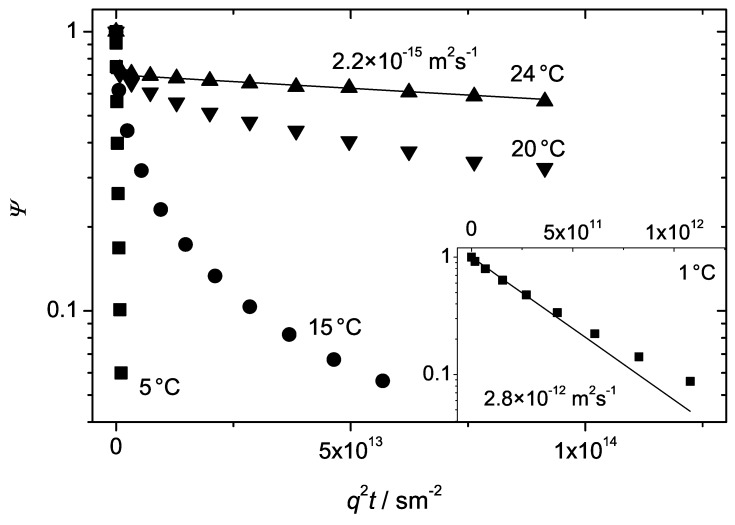
The normalized spin echo diffusion attenuations of the triblock copolymer P123 in water at c=0.29 for four different temperatures. The inset shows the attenuation curve at $1{\;}^{\circ }$C.

The increase of the polymer concentration of the investigated samples in water above c>0.25 results in the formation of the liquid crystalline cubic phase. The diffusion attenuation curves at room temperature in the cubic phase are shown in Figure 4 (curves 7 and 8) for P123 and in Figure 5 (curves 8 and 9) for P104. Figure 6 shows in addition the evolution of the attenuation curves for P123 with c=0.29 at temperatures below the ambient.

Figure 4 and Figure 5 demonstrate a radical decrease of the initial slopes of the attenuations in comparison to the micellar phase (compare, for instance, the curves 7 and 8 with the curve 1 in Figure 4 or the curves 8 and 9 with the curve 1 in Figure 5). Besides, in contrast to the micellar phase, the attenuation curves exhibit considerable deviations from the exponential behaviour. The observed departure from exponentiality should be attributed to the distribution of the diffusion coefficients. The attenuations observed in the cubic phase were extremely weak in spite of very high magnetic field gradients applied in our experiments. The maximal attenuation of the initial echo amplitude (achieved within the technical limitations) was insufficient for the reliable quantitative analysis of the measured curves in terms of the distribution functions, see for instance curves 7 and 8 in Figure 4 and curves 8 and 9 in Figure 5. However, rough estimates of the mean diffusivity can be given for the dominating part of molecules.

A closer inspection of the curves showed that they can tentatively be decomposed into two components:

(5) $$\Psi \left(q,t\right)=\Psi_{fast}\left(q,t\right)+\Psi_{slow}\left(q,t\right)$$

where $\Psi_{fast}\left(q,t\right)$ and $\Psi_{slow}\left(q,t\right)$ refer to the faster (initial part) and the slower components of the attenuations. We cannot specify whether any of the components follows the monoexponential function and assume a more general multiexponential behaviour, Equation (2), with different distribution functions for both $\Psi_{fast}\left(q,t\right)$ and $\Psi_{slow}\left(q,t\right)$. The relative fractions of the fast component are less than 20% for the curves 7 and 8 in Figure 4 and less than 30% for the curves 8 and 9 in Figure 5. The initial slopes exceeded $10^{-14}$ m${}^{2}$s${}^{-1}$ but could not be evaluated more precisely. The presence of the faster attenuating components (and so of the species with larger diffusivities) may arise due to several factors. The latter include the imperfections in the cubic crystalline formation (“loose” molecules not trapped in the ordered structure, defects in the micellar packing order), the coexistence of the micellar liquid and cubic phases, the presence of diblock and homopolymer impurity fractions [6], *etc*. These factors could not be identified without a more detailed investigation, which was beyond the scope of this work.

The component of interest in this case was the slower one, which refers to the dominating fraction of molecules in both samples (that is, more than 80% in P123 and more than 70% in P104). We attribute this component to the cubic crystalline formation itself. Assuming that $\Psi_{slow}\left(q,t\right)$ follows Equation (2), the mean diffusivity $\bar{D}$ of the slow molecular fraction can be evaluated according to Equation (3) with P(D) related to this fraction only. The estimated values of $\bar{D}$ in P123 and P104 were about $3\times 10^{-15}\;$ m${}^{2}$s${}^{-1}$ and $8\times 10^{-15}\;$ m${}^{2}$s${}^{-1}$, respectively. The corresponding slopes are shown by the solid lines in Figure 4 and Figure 5.

The above estimations indicate that diffusion in the cubic phase is dramatically retarded. The difference between the values of $\bar{D}$ and the diffusivities in the micellar phase is very large. For example, the diffusion coefficient of P123 in the micellar phase with c=0.24 is $2\times 10^{-12}\;$ m${}^{2}$s${}^{-1}$. The latter is nearly three orders of magnitude larger than $\bar{D}$ in the cubic phase which occurs at just a few weight percents larger concentration, c=0.28. This radical change of the diffusivities within a narrow concentration range is obviously the result of the transition from the micellar liquid to the micellar ordered cubic crystalline phase with closely packed micelles. The existence of the cubatic order was demonstrated for various Pluronics with different molecular weights and block compositions with the help of a SANS study [9,32]. The scattering patterns observed indicated the arrangement of spherical micelles into a body-centered cubic lattice.

SANS studies have also shown that for moderate polymer concentrations the micellar volume fraction tends to linearly increase with increasing *c* [9]. The transition from the micellar suspension to the cubic crystal occurs at concentrations above ∼0.2 (the exact value depends on the BCP composition and mass), at which the micellar volume fraction reaches the limiting value of ϕ=0.53 (the critical value of the hard-sphere crystallization [32,33]). The solid-like gel is formed accompanied by dramatic changes of rheological properties. The observed radical retardation of diffusion in our samples is in agreement with these findings.

A remarkable feature of the Pluronic cubic phases is associated with the so-called “inverse melting”, that is, with melting the ordered lattice arrangement when decreasing the temperature. Temperature produces a crucial effect on the diffusion behaviour in the cubic phase. This is shown in Figure 6 for P123 with c=0.29. As temperature decreases below $20{\;}^{\circ }$C, the initial slope of the attenuations decreases jump-like and the deviations from the exponential behavior tend to vanish. At $1{\;}^{\circ }$C, only slight deviations from the monoexponential function were observed, see the inset in Figure 6. The diffusivities evaluated from the initial slopes become comparable to that in the micellar phase, $D>10^{-12}\;$ m${}^{2}$s${}^{-1}$, compare with the data in Figure 3.

The maximal molecular displacements during the observation time corresponding to the slow diffusion modes in the cubic phase are in the range of 50 to 100 nm. The hydrodynamic radius of micelles depends on the molecular block composition and temperature and is typically in the range of a few to ten nanometers [10]. The reported values [6] of the micellar radii in P123 and P104 (at room temperature in diluted solutions) evaluated from the light scattering experiments are 3.2 nm and 5.8 nm, respectively. Molecular displacements monitored in our experiments are considerably larger than the characteristic micellar sizes and indicate long-range diffusion.

**Figure 7:**
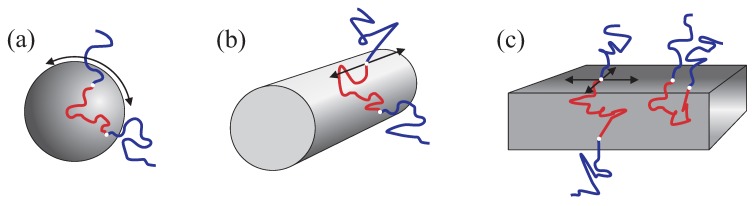
Schematic presentation of possible diffusion displacements of the triblock copolymer chains in micellar (**a**) rod-like cylindrical (**b**) and lamellar (**c**) substructures. The arrows indicate the directions of unrestricted interface diffusion.

The following molecular diffusion mechanisms are generally possible with respect to cubic micellar arrangements: (a) interface diffusion along the boundary of the spherical micelle, see Figure 7a; (b) chain exchange between the micelles; (c) diffusion of the entire micelles similar to that in the liquid micellar phases. Since micelles form closed entities, the interface diffusion cannot contribute to molecular displacements exceeding the typical micellar size. Therefore, the mechanisms (b) and (c) are the only ones that may potentially contribute to the long-range diffusion. The mechanism (b) was reported as the probable mechanism [11,18] in BCP melts and cannot be excluded for our samples too. Diffusion of entire micelles (c) is also a likely mechanism. One can assume that the three-dimensional cubatic crystalline order in BCP mixtures with solvents is far from being perfect and contains structural defects in form of “empty” unoccupied sites. These defects can enable very slow migration of the entire micelles from the one “empty” site to another. If this is the dominating mechanism in our samples, the diffusion coefficient related to such migration will be 2–3 orders of magnitude smaller than in the micellar liquid state, which seems to be a reasonable value.

It turns out that the cubic phase exhibits the slowest diffusion attenuations not only in comparison to the micellar liquid but also in comparison to the other two phases, the hexagonal and the lamellar ones, formed at higher polymer concentrations. The latter phases are discussed in more detail below.

### 3.3. The Hexagonal and the Lamellar Phases

With increase of the concentration above 0.45 for P123 and 0.5 for P104, the hexagonal mesophase forms. It consists of the rod-like cylindrical structures packed with a hexagonal symmetry, see the schematic drawing in Figure 1c. Further increase of *c* above ∼0.7 corresponds to the transition to the lamellar mesophase (Figure 1d) in both Pluronic samples.

The attenuation curves in the hexagonal phase are shown for three different concentrations in Figure 4 (curves 4–6) and Figure 5 (curves 5–7). The lamellar phase is represented by curves 2 and 3 in Figure 4 and curves 2–4 in Figure 5. The concentration effects within the same phase are minor. However, the initial slopes and the shapes of the curves in the hexagonal and the lamellar phases differentiate dramatically from each other and from those observed in the micellar and cubic phases. The initial slopes of the curves in the hexagonal phase range between those of the cubic and the lamellar phases. Note a pronounced non-exponentiality of the curves in both samples. A transition from the hexagonal to the lamellar phase is accompanied by an essential drop in the initial slopes, compare the curves 2 and 3 with the curves 4–6 in Figure 4 and the curves 2–4 with the curves 5–7 in Figure 5. Also, a distinguishable decrease of the non-exponentiality following the transition to the lamellar phase was observed in both P123 and P104.

Pronounced differences in the behavior of the diffusion attenuations in the anisotropic mesophases (hexagonal and lamellar) and in the micellar isotropic phases (disordered liquid and cubic) should be analysed in terms of their molecular architectures. The microstructure formation with segregated blocks in BCPs occurs through localization of inter-block junctions in the more or less sharp interface regions. The latter separate the domains composed predominantly of one of the blocks. The BCP molecules can diffuse along the interfaces without perturbing the domain segregation. In contrast to the discrete micellar aggregates in the isotropic solutions or gels, domain structures in the anisotropic mesophases (cylinders or lamellae) are extended in space. This provides a macroscopic connectivity enabling the long-range diffusion of individual BCP molecules.

In the following, diffusion of P123 in the hexagonal and in the lamellar phases will be analysed in terms of the model for anisotropic diffusion [34,35] in the randomly oriented array of elements. Each element is assumed to posses a cylindrical symmetry and no molecular exchange between the elements is anticipated. Two diffusion coefficients are distinguished, $D_{∥}$ and $D_{⊥}$, describing molecular displacements parallel and perpendicular to the element director, respectively. Averaged over all possible orientations of the array elements, the diffusion attenuation is described according to [35]:

(6) $$\Psi =\exp\left(-kD_{⊥}\right)\int_{0}^{1}\exp\left[-k\left(D_{∥}-D_{⊥}\right)\;x^{2}\;\right]\;dx$$

where $k=q^{2}t$.

If diffusion in direction across the symmetry axis is largely hindered, that is, $D_{∥}\gg D_{⊥}$, Equation (6) reduces to the one-dimensional case:

(7) $$\Psi_{1D}=\int_{0}^{1}\exp\left[-kD_{∥}\;x^{2}\;\right]\;dx$$

In the opposite case of two-dimensional (lamellar) diffusion the attenuation is described as:

(8) $$\Psi_{2D}=\exp\left(-kD_{⊥}\right)\int_{0}^{1}\exp\left[kD_{⊥}\;x^{2}\;\right]\;dx$$

**Figure 8:**
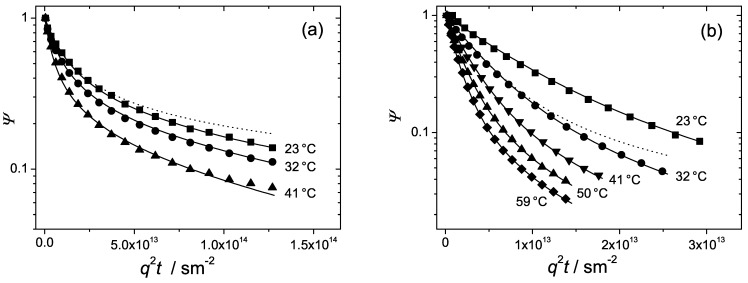
The normalized spin echo diffusion attenuations of the triblock copolymer P123 in water at different temperatures in the (**a**) hexagonal phase, c=0.5 and (**b**) lamellar phase, c=0.7. Solid curves are fits of Equation (6) to the data points. The dashed curves are fits of the (**a**) Equation (7) in the “one-dimensional” limit and (**b**) Equation (8) in the “two-dimensional” limit.

The diffusion attenuations observed in the hexagonal and lamellar phases are shown once again in Figure 8 together with the fits of Equation (6), solid curves. Excellent fits were obtained. The only fit parameters were $D_{∥}$ and $D_{⊥}$. The dashed curve in Figure 8a represents the fit of Equation (7) with a single fit parameter, $D_{∥}$ to the experimental points at $23{\;}^{\circ }$C. The dashed curve in Figure 8b is the fit of Equation (8) with the fit parameter, $D_{⊥}$ to the experimental curve at $32{\;}^{\circ }$C. The fitted values of $D_{∥}$ and $D_{⊥}$ and the calculated values of the two anisotropy factors, $D_{∥}/D_{⊥}$ and $D_{⊥}/D_{∥}$ are represented in Table 2. For convenience, for characterizing diffusion anisotropy in the hexagonal or lamellar phases we will use that of the parameters, $D_{∥}/D_{⊥}$ or $D_{⊥}/D_{∥}$, which will have the bigger value.

**Table 2:** The diffusion coefficients evaluated from the fits of Equation Equation (6) to the experimental data represented in Figure 8.

Morphology	*T*	$D_{∥}$	$D_{⊥}$	$D_{∥}/D_{⊥}$	$D_{⊥}/D_{∥}$
	[°C]	[$m^{2}\;s^{-1}$]	[$m^{2}\;s^{-1}$]		
hexagonal	23	$2.1\times 10^{-13}$	$1.7\times 10^{-15}$	124	$8.1\times 10^{-3}$
hexagonal	32	$2.8\times 10^{-13}$	$2.4\times 10^{-15}$	117	$8.6\times 10^{-3}$
hexagonal	41	$5.2\times 10^{-13}$	$3.8\times 10^{-15}$	137	$7.3\times 10^{-3}$
lamellar	23	$1.5\times 10^{-14}$	$1.7\times 10^{-13}$	$8.8\times 10^{-2}$	11
lamellar	32	$2.0\times 10^{-14}$	$3.1\times 10^{-13}$	$6.4\times 10^{-2}$	16
lamellar	41	$2.4\times 10^{-14}$	$5.0\times 10^{-13}$	$4.8\times 10^{-2}$	21
lamellar	50	$2.6\times 10^{-14}$	$7.3\times 10^{-13}$	$3.6\times 10^{-2}$	28
lamellar	59	$2.7\times 10^{-14}$	$1.0\times 10^{-12}$	$2.7\times 10^{-2}$	37

Table 2 shows that in the hexagonal phase $D_{∥}\gg D_{⊥}$. This suggests that diffusion perpendicular to the rod director is strongly retarded. The anisotropy factor, $D_{∥}/D_{⊥}$, is remarkably large, in the range of 120–140. Such a large anisotropy permits one to interpret diffusion in the hexagonal phase as quasi one-dimensional. The dashed curve in Figure 8a is the fit of Equation (7) to the data points in the strictly one-dimensional limit. As one can see, the departure of the fit from the experimental curve is not considerably big, which complies with the above conclusion.

In the lamellar phase, in contrast, lateral diffusion appears to be much faster than diffusion parallel to the element director, $D_{⊥}\gg D_{∥}$. The anisotropy factor $D_{⊥}/D_{∥}$ is around 10. This is smaller than the corresponding anisotropy factor in the hexagonal phase. Still, lateral diffusion along the interface is by one order of the magnitude faster than that in the perpendicular direction and represents thus the dominating mechanism. Therefore, diffusion in the lamellar phase can be regarded as quasi two-dimensional. The dashed curve in Figure 8b is the fit of Equation (8) in the two-dimensional limit. Again, we see that the deviations from the the experimental curve at $23{\;}^{\circ }$C are not too big in agreement with the observed anisotropy factor.

It is worth noting that the values of $D_{∥}$ in the hexagonal phase and the values of $D_{⊥}$ in the lamellar phase are very close to each other. This indicates that the intrinsic properties of molecular diffusion in both formations are largely the same. The main difference refers to the anisotropy and different dimensionality of diffusion: quasi one-dimensional in hexagonal and quasi two-dimensional in the lamellar structure. Note that according to the applied model diffusion within a given element is assumed to be normal. The observed strong non-exponentiality of the diffusion curves is solely due to powder-like orientation of the supramolecular structures within the sample. No dependence of the attenuations on the observation time in the range below 400 ms was observed. The corresponding mean square molecular displacements are in the range of 500 nm. The latter value gives an estimate of the smallest length scale where the domain structure orientation is homogeneous. In general, one can conclude that diffusion in hexagonal and lamellar structures tends to be quasi one- or two-dimensional, in analogy with the properties of liquids confined in narrow cylindrical or slit-like pores.

### 3.4. Effects of Self-Assembly on Molecular Diffusion as A Function of Polymer Concentration

Figure 4 and Figure 5 show that the concentration behaviour of the attenuation curves of the investigated BCPs in water sharply contrasts to that of soluble polymers and oligomers. Typically, the attenuations in monodisperse polymer solutions of relatively low molecular mass are exponential (isotropic diffusion) and the diffusivities decrease smoothly as a function of concentration. The example was already shown for PEO water solutions in Figure 2 and Figure 3. In the studied BCP samples, the initial slopes change jump-like with increasing concentration indicating phase transitions between the different structure formations. Besides, the attenuation curves exhibit essentially different shapes for various phases.

**Figure 9:**
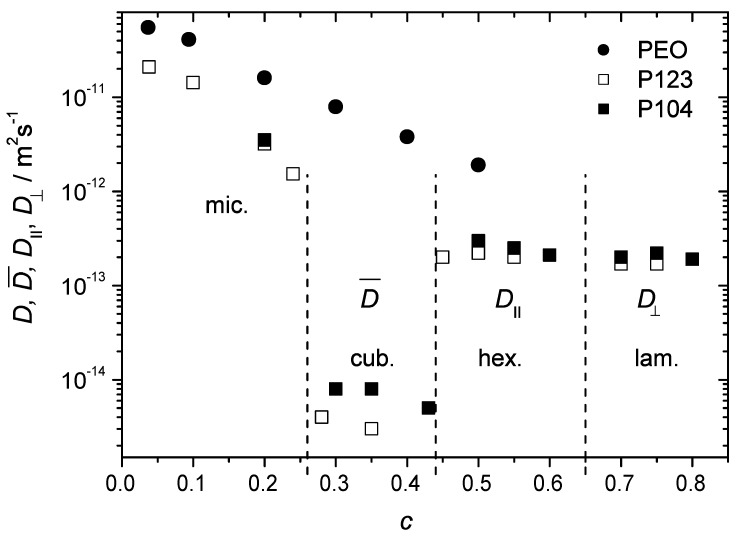
The diffusion coefficients of polymer molecules in the samples P123, P104 and PEO in water as a function of polymer concentration in the broad range from 0.1 to 0.8 at $T=23{\;}^{\circ }$C.

Figure 9 shows the diffusion coefficients of the studied polymers as a function of *c* covering a broad concentration range, from 0.09 to 0.8. Here, the data points in PEO water solutions and in the BCP micellar liquid state refer to a single diffusion coefficient characteristic of the majority of molecules. The hexagonal and the lamellar phases are represented by the values of $D_{∥}$ and $D_{⊥}$, respectively. The cubic phase is represented by the mean diffusivity of the dominating slow molecular fraction (above 70% of molecules). Each phase can be distinguished on the basis of a characteristic diffusion behavior. In the micellar phase, *D* decreases with increasing *c* as in homopolymer solutions. The transition to the cubic phase is accompanied by a dramatic drop of the diffusivity below $10^{-14}\;$ m${}^{2}$s${}^{-1}$. Anisotropic mesophases are characterized by strongly anisotropic diffusion with $D_{∥}$ and $D_{⊥}$ dominating in the hexagonal and lamellar phases, respectively. The data in Figure 9 thus permit to conclude that diffusion properties of BCP molecules in various structure formation can be used as a complementary means for the construction of the phase diagrams.

Diffusion mechanisms of BCPs in anisotropic mesophases were approached in Refs. [11,18] with respect to melts. The results of melt studies cannot be directly compared to that in aqueous mesophases, of course. However, the influence of constraints due to domain segregation on the intrinsic diffusion mechanisms will be similar in melts and liquid crystal mesophases. The main mechanisms in question are the “interface diffusion” and the “hopping diffusion”. The interface diffusion is assumed to be the dominating diffusion mechanism and refers to molecular displacements along the boundary layers separating the domains with a predominant localization of only one block, see Figure 7. The boundary layer contains the interblock junctions and may be sharp or loose, depending on segregation conditions. Diffusion of BCP molecules along the interface does not disrupt localisation of microdomains and of interface junctions. Therefore, it is not subjected to thermodynamic penalty. This is in contrast to the “hopping diffusion”, which is characteristic for diffusion of the chains from one microstructure unit to another. That is, this mechanism anticipates that BCP molecules “exchange” between different domains in spite of the energetic barriers (activation process). This mechanism allows diffusion in direction perpendicular to the cylinder axis in the hexagonal or to the lamellar plane in the lamellar phases. Our findings indicate that the main diffusion mechanism is interface diffusion whereas hopping diffusion is strongly retarded.

## 4. Summary and Conclusions

Diffusion of triblock copolymers, PEO-PPO-PEO, has been studied in a broad range of polymer concentrations in water. The system exhibits a variety of super-molecular structures based on molecular self-assembly. By imposing internal constraints on molecular movements, structure formation produces dramatic “pore-like” effects determining the properties of molecular diffusion. It occurs that, in mixtures with water, diffusion of the studied Pluronic samples is governed mainly by the type of ordering in different structure morphologies rather than by typical steric hindrances and excluded volume effects as in the solutions of their homopolymeric counterpart (PEO). Furthermore, diffusivities did generally not depend on the observation time, suggesting unrestricted (albeit hindered) diffusion for all concentrations studied. In particular micelle suspensions with polymer weight fractions below 0.2 exhibit normal (Gaussian) diffusion. Diffusivities smoothly decrease with increasing concentration, which is typical also for the homopolymer solutions. The reduction of diffusion coefficients in comparison to that of PEO molecules in water solutions is a consequence of the larger effective mass and size of diffusing entities (micelles *versus* polymer coils). Micelles are characterised by rather uniform sizes. Formation of cubic crystals at higher concentrations (rationalised within the model of the “hard sphere crystalization”) results in an extreme retardation of diffusion. The estimated mean diffusivity of the dominating part of molecules is below $10^{-14}$ m${}^{2}$s${}^{-1}$. A sharp increase of diffusivities by 2–3 orders of magnitude during melting of the cubic crystal below $15{\;}^{\circ }$C suggests that again liquid micellar or unimer solution is formed. In the hexagonal and lamellar structures diffusion is strongly anisotropic due to constraints imposed by specific molecular ordering on the molecular displacements. Structural restrictions are so efficient that diffusion occurs to be quasi one-dimensional, parallel to the director of hexagonally aligned rods, or it is lateral (quasi two-dimensional) along lamellar interfaces. Referred to these directions, diffusion remains normal and unrestricted on a length scale of at least 500 nm. The diffusivities along the respective domain interfaces are nearly the same for both hexagonal and lamellar structures, indicating that molecular organization on the short local length scale (essential for intrinsic diffusion properities) is similar in both phases. The main differences are rather due to reduced dimensionality of diffusion caused by a specific type of ordering on the mesoscopic lengths scale. The dominating diffusion mechanism in the anisotropic mesophases was shown to be the “interface diffusion”.
